# Equine Piroplasmosis Associated with *Amblyomma cajennense* Ticks, Texas, USA

**DOI:** 10.3201/eid1710.101182

**Published:** 2011-10

**Authors:** Glen A. Scoles, H. Joel Hutcheson, Jack L. Schlater, Steven G. Hennager, Angela M. Pelzel, Donald P. Knowles

**Affiliations:** US Department of Agriculture and Washington State University, Pullman, Washington, USA (G.A. Scoles, D.P. Knowles);; US Department of Agriculture, Ames, Iowa, USA (H.J. Hutcheson, J.L. Schlater, S.G. Hennager);; US Department of Agriculture, Fort Collins, Colorado, USA (A.M. Pelzel)

**Keywords:** equine piroplasmosis, tick-borne diseases, vector-borne infections, Theileria equi, Amblyomma cajennense, equids, equine diseases, horses, parasites, ticks, Texas, expedited, dispatch

## Abstract

We report an outbreak of equine piroplasmosis in southern Texas, USA, in 2009. Infection prevalence reached 100% in some areas (292 infected horses). *Amblyomma cajennense* was the predominant tick and experimentally transmitted *Theileria equi* to an uninfected horse. We suggest that transmission by this tick species played a role in this outbreak.

*Theileria equi* (*incertae sedis*; *Piroplasma equi* Laveran, 1901) is one of the etiologic agents of equine piroplasmosis. This parasite infects equids worldwide, but a few countries (Australia, Great Britain, Japan, United States, and Canada) are classified as free of this disease. These and several other countries restrict entry or internal movement of horses on the basis of their serologic response to *T. equi* antigen.

Particular tick species are obligate intermediate hosts and vectors for *T. equi* (*1*), which undergoes a complex developmental cycle in the vector similar to that of other apicomplexan hemoparasites (*2**,**3*). Asymptomatic persistent parasitemia detectable by serologic analysis or PCR develops in equids that survive acute infection. International movement of asymptomatic carriers poses a risk for introduction of equine piroplasmosis into regions free of this disease, but endemic transmission occurs only in regions that have competent vectors.

The World Organisation for Animal Health has listed the United States as free of equine piroplasmosis since 1978, although recent cases have occurred. Some of these cases may have resulted because the complement fixation test, formerly used for import screening, was not sufficiently sensitive to make a correct diagnosis. When transmission has occurred, it has been iatrogenic rather than vector-borne. Only 2 experimentally competent vectors of *T. equi* are known in the United States: *Dermacentor variabilis* (American dog tick) and *Rhipicephalus* (*Boophilus*) *microplus* (southern cattle tick) (*4*). However, of the 90 tick species in the United States, few have been tested for equine piroplasmosis vector competence (*1**,**4*).

## The Study

On October 2, 2009, a mare in Kleberg County, Texas, USA, showed clinical signs of equine piroplasmosis. Serologic testing at the Animal Plant Health Inspection Service, National Veterinary Service Laboratories (NVSL), US Department of Agriculture (USDA) (Ames, IA, USA) with a commercially available competititve ELISA (VMRD Inc., Pullman, WA, USA) detected *T. equi* antibodies. The remaining 359 horses on the index ranch were tested in the same way, and 292 (81.1%) of 360 were seropositive for *T. equi* on initial screening (Table 1).

**Table 1:** Horses tested by competitive ELISA for *Theileria equi* on index ranch of equine piroplasmosis outbreak in southern Texas, USA, 2009

Ranch division	No. positive/no. tested (%)
A*	213/281 (75.8)
B	36/36 (100)
C	10/10 (100)
D	33/33 (100)
Total	292/360 (81.1)

*Division A contained all younger stock. Infection rates among younger animals were lower. The other 3 divisions contained mostly horses used for working cattle.

Ticks collected from horses on the index ranch were shipped alive to NVSL. Identifications were made by using morphologic characteristics, geographic distribution, biologic characteristics, and host associations (*5**–**9*). NVSL received ticks from 228 horses; >1 species was present on 41 animals. The dominant tick, *Amblyomma cajennense*, was collected from 180 (78.9%) horses (Table 2).

**Table 2:** Tick species found on horses at index ranch of equine piroplasmosis outbreak in southern Texas, USA, 2009

Species	No. (%) horses*	No. ticks			Average no. ticks/horse
		Male	Female	Nymph	
*Amblyomma cajennense*	180 (78.9)	201	229	1	2.4
*A. maculatum*	45 (19.7)	43	33	0	1.7
*Dermacentor* (*Anocentor*) *nitens*	7 (3.0)	4	7	3	2
*D. variabilis*	37 (16.2)	20	34	0	1.6

*Of 228 horses sampled, 41 had >1 species of tick present.

All ticks were identified and sent to the Agricultural Research Service, Animal Disease Research Unit, USDA (Pullman, WA, USA) for transmission studies. Live males and partially fed females were pooled by species and held at 25°C and a relative humidity of 98% until they were allowed to reattach and feed on uninfected horses.

A total of 104 *A. cajennense* ticks (45 male and 79 female) were placed on a horse on October 30, 31, and November 2, 2009. These ticks had been removed from 73 horses on the index ranch, of which 68 (93.2%) were seropositive for *T. equi*. Females were allowed to reattach and feed until repletion; males were removed when all females were replete. All ticks were removed by November 18, 2009. Twenty-four fully engorged females and 3 live males were recovered. The horse had a fever (>39°C) 14 days after the ticks were first applied. Parasitized erythrocytes on a stained blood smear peaked at 0.3% on day 17. No other clinical signs of infection were evident. Serologic analysis and PCR (*10*) confirmed *T. equi* infection.

Twenty-nine *D. variabilis* ticks (12 male and 17 female) were placed on a second uninfected horse on October 30 and 31 and November 2 and 12, 2009. These ticks had been removed from 17 horses, of which 11 were seropositive and 1 was seronegative for *T. equi*; 5 had an unknown infection status. All ticks were removed by November 24, 2009. Six fully engorged females and 7 live males were recovered. This horse had a slight fever (39°C) 15 days after tick attachment but otherwise showed no clinical signs. No organisms were found in blood smears, but this horse was positive for *T. equi* by PCR 42 days after the first ticks were attached and by competitive ELISA 87 days after tick attachment.

## Conclusions

Ranch staff reported that they used no practices that would result in movement of blood-contaminated materials between horses (e.g., no reuse of needles), which suggests that iatrogenic transmission was not responsible for this outbreak. Consequently, the high prevalence of *T. equi* infection implies a focus of vector-borne transmission.

*A. cajennense* ticks were the most abundant species on horses during the period (October–November) of this investigation (Table 2). Our results demonstrate that *A. cajennense* ticks naturally acquired infection while feeding on infected horses and transmitted *T. equi* intrastadially when they reattach and feed on uninfected hosts. *A. cajennense* ticks have not been shown experimentally to be a competent vector for *T.* *equi* (*1*). This species is a 3-host tick, and all life stages are known to feed aggressively on a wide variety of hosts, including horses. The natural distribution of *A. cajennense* ticks includes southeast Texas; they are not known to be present in other parts of the United States where cases of equine piroplasmosis have occurred (*11*). Although this study demonstrates that *A. cajennense* ticks are an experimental intrastadial vector, additional studies are needed to fully characterize the vector capacity of this species, particularly with regard to interstadial transmission.

Immature stages of *D. variabilis* ticks occur almost exclusively on rodents; only adults were found on horses at the ranch. Although these ticks were able to transmit *T.* *equi* intrastadially to an uninfected horse, the small proportion of infested horses on the ranch (16.2%; Table 2) and low transmission efficiency of this species (G.A. Scoles, unpub. data) make it unlikely that *D. variabilis* ticks were responsible for the high infection prevalence.

*A. maculatum*, the Gulf Coast tick, was the second most abundant species on horses at the index ranch during the study (19.7%; Table 2). However, this species survived poorly during handling and transport, probably because it is less tolerant of desiccation than are *A. cajennense* ticks (*12*), and we did not have enough viable ticks to attempt transmission feeding. Whether this species can act as a vector for *T. equi* is unknown. *D*. (*Anocentor*) *nitens* ticks were collected from 7 (3%) horses sampled. This species is a proven vector of *Babesia caballi* but has not been shown to be a vector of *T. equi*. *R. microplus* ticks are limited to a quarantine zone along the Texas–Mexico border. The index ranch is north of this zone, and no *R. microplus* ticks were found on horses at this ranch.

Although *T. equi* can be transmitted iatrogenically, e.g., by common needle use (*13*), this route of transmission is improbable with good management practices. Vector-borne transmission is more likely than iatrogenic transmission to establish and maintain a large focus of infection, such as in this outbreak. Additional tick studies are needed to determine whether other indigenous tick species are involved in transmission at this site. However, if *A. cajennense* ticks are the primary vector, the outbreak will likely be confined to this region because southeastern Texas is the northern extent of the range of this tick in the United States (*11*). Given knowledge of tick species that are competent vectors, spread of this parasite can be controlled by testing requirements and limits on regional movement of equines on the basis of presence or absence of such competent vectors.
